# Predation by Native Mediterranean Species on the Invasive Blue Crab: Experimental Evidence from the Common Octopus and the Loggerhead Sea Turtle

**DOI:** 10.3390/ani15243566

**Published:** 2025-12-11

**Authors:** Silvia Falco, Miguel Rodilla, José Luis Crespo-Picazo, Daniel García-Párraga, Ignasi Gairin, Patricia Prado

**Affiliations:** 1 Instituto de Investigación para la Gestión Integrada de Zonas Costeras (IGIC), Universitat Politècnica de València, C/Paranimf 1, 46730 Gandía, Valencia, Spain; mrodilla@upv.edu.es; 2 Fundación Oceanogràfic de la Comunitat Valenciana, C/Eduardo Primo Yúfera (Científic), 1B, 46013 Valencia, Valencia, Spain; jlcrespo@oceanografic.org (J.L.C.-P.); dgarcia@oceanografic.org (D.G.-P.); 3 Institut de Recerca i Tecnologia Agroalimentàries (IRTA-La Ràpita), Ctra. Poble Nou Km 5.5, 43540 Sant Carles de la Ràpita, Tarragona, Spain; ignasi.gairin@irta.cat (I.G.); patricia.prado@ucv.es (P.P.); 4 Instituto de Investigación en Medio Ambiente y Ciencia Marina (IMEDMAR-UCV), Universidad Católica de Valencia SVM, C/Explanada del Puerto S/n, 03710 Calpe, Alicante, Spain; 5 Institut d’Estudis Professionals Aqüícoles i Ambientals de Catalunya (IEPAAC), 43540 Sant Carles de la Ràpita, Tarragona, Spain

**Keywords:** invasive species, *Callinectes sapidus*, predation, loggerhead sea turtle, common octopus, biological control

## Abstract

The rapid spread of the invasive blue crab (*Callinectes sapidus*) in Mediterranean coastal ecosystems poses ecological and socio-economic challenges, highlighting the need to explore natural control mechanisms. We conducted controlled aquarium experiments to assess the functional size preference and feeding rates of two native Mediterranean predators: the common octopus (*Octopus vulgaris*) and the loggerhead sea turtle (*Caretta caretta*). Two sets of trials were performed: (1) size preference experiments, in which individual predators were simultaneously offered crabs of varying sizes; and (2) ad libitum feeding experiments to estimate daily consumption rates. Octopuses consumed only the soft tissues biomass (STB, ~54% of total crab biomass), reaching maximum intake rates of 454 ± 71 gWW·d^−1^ in adult individuals. Sea turtles ingested whole crabs, including the exoskeleton, with total consumption rates up to 815 ± 592 gWW·d^−1^ of total biomass (TB) in adults. Subadult octopuses exhibited shorter handling times for small crabs, although neither predator showed a statistically significant size preference. Our findings indicate that both predators can consume substantial amounts of blue crab biomass, but *O. vulgaris*—due to its higher feeding efficiency and ecological abundance—might play a more prominent role as a natural biocontrol agent. These results provide valuable insights for integrated management strategies aimed at mitigating the ecological impact of *C. sapidus* in the Mediterranean.

## Simple Summary

The blue crab is an invasive species that has rapidly spread along Mediterranean coasts, threatening marine ecosystems. Exploring natural ways to control its population could help reduce these impacts. In this study, we investigated the functional size preference and feeding rates of two native predators—the common octopus and the loggerhead sea turtle—when feeding on blue crabs. In aquarium experiments, predators were offered crabs of different sizes under controlled conditions to assess whether they preferred certain sizes and how much they could consume in a day. Both species consumed substantial amounts of blue crabs: octopuses fed only on soft tissues, whereas turtles ingested whole crabs, including the exoskeleton. Octopuses exhibited shorter handling times for small crabs, although neither predator showed a marked size preference. Our findings suggest that, particularly octopuses, thanks to their abundance and feeding efficiency, could contribute to limiting invasive crab populations and support ecosystem balance.

## 1. Introduction

Biological invasions represent one of the main threats to biodiversity, ecosystem functioning, and fisheries sustainability in coastal environments worldwide [1]. In the Mediterranean Sea, the Atlantic blue crab (*Callinectes sapidus* Rathbun, 1896)—a portunid native to the Atlantic coasts of the Americas—has become one of the most conspicuous invasive species [2]. First reported in the Northern Adriatic Sea in 1949 [3], its expansion has accelerated over the past decades, with established populations now widely distributed across the western Mediterranean basin [2,4].

This species is characterized by a complex life cycle that spans a wide salinity gradient. Reproduction occurs in oligohaline zones, after which ovigerous females migrate to polyhaline coastal waters to spawn, ensuring larval development in high-salinity conditions [5,6]. Following a planktonic phase at sea, megalopae and juveniles actively recruit back into estuarine nurseries [7]. This life strategy enhances the ecological plasticity and colonization success of *C. sapidus*, which can exert substantial impacts at multiple trophic levels in invaded ecosystems [2] from fish to a diversity of invertebrate prey [8], particularly mollusks, some with paramount commercial interest [9].

In its native range, top-down control by natural predators plays an essential role in regulating blue crab populations [10]. However, in the Mediterranean Sea, trophic interactions involving this invasive species remain poorly documented. Recent observations indicate that some native Mediterranean predators have incorporated *C. sapidus* into their diets, suggesting an adaptive response to its increasing availability. In marine habitats, predation has been reported by rays [11], the common octopus (*Octopus vulgaris*) [12,13], and the loggerhead sea turtle (*Caretta caretta*) [14]. Conversely, in freshwater and oligohaline systems, few documented records exist of adult blue crab predation, with only isolated reports of crab remains left by Eurasian otters (*Lutra lutra*) [15].

The common octopus (*O. vulgaris*) is a widespread, semelparous cephalopod species that inhabits coastal waters of the Mediterranean, typically from 0 to 60 m depth [16,17]. It displays a flexible and opportunistic feeding behavior, primarily consuming crustaceans, mollusks, and fish [18,19,20]. Prey selection in this species is known to be influenced by habitat complexity and prey availability [21], with seasonal changes in feeding intensity often associated with temperature shifts [22]. Beyond these factors, body size also plays a crucial role in shaping predator–prey interactions, as it is closely linked to morphology, physiology, and behavior, and is increasingly recognized as a key determinant of ecological performance and trophic dynamics [23].

The loggerhead sea turtle (*C. caretta*) is the most abundant sea turtle species in the Mediterranean [24]. Its life cycle includes ontogenetic habitat shifts from oceanic to neritic environments, accompanied by a dietary transition from pelagic to benthic prey [25,26]. However, recent studies suggest an early recruitment to shallow coastal zones and a consistent predatory strategy across life stages, with crustaceans and mollusks as major prey components [14,27]. Notably, the western Mediterranean has experienced an increase in loggerhead turtle nesting in recent years [28], a trend potentially favored by climate-driven thermal changes [29].

Despite increasing observational evidence, the actual predation rates and trophic impacts of *O. vulgaris* and *C. caretta* on invasive blue crab populations remain largely unknown. Manipulative experiments can provide mechanistic insights into predator-prey interactions and evaluate the potential for natural biocontrol in invaded systems [30]. Here, we conducted controlled laboratory experiments to assess size preference and feeding rates of the common octopus and the loggerhead sea turtle when preying on blue crabs. As noted by Singer [31] and Underwood et al. [32], predator–prey interactions depend on both preference (a predator trait) and acceptability (a prey property). Because distinguishing these components would require both choice and no-choice trials, which are impractical with sensitive complex organisms and protected marine species, our results are interpreted as functional preferences, shaped by the behavior of predators and the traits of their prey. Therefore, this study aimed to assess, under controlled laboratory conditions, the functional size preference and the feeding rates of two native Mediterranean predators—the common octopus and the loggerhead sea turtle—to explore their potential role in the biocontrol of the invasive blue crab within Mediterranean ecosystems.

## 2. Materials and Methods

### 2.1. Collection and Maintenance of Organisms

Experimental trials were carried out under controlled laboratory conditions to test the interaction between two native Mediterranean predators and the invasive blue crab.

Octopuses were captured using authorized traps in the outer sea area of the Ebro Delta (Western Mediterranean coast, Spain), near the river mouth. Subadult individuals (O_SA, ca. 1 kg) were collected in late October 2021, and adult individuals (O_A, ca. 2 kg) were collected in late March 2022. Size classification followed González et al. [33], based on body weight and reproductive status. All octopuses were transported in aerated tanks of approximately 250 L to the IRTA (Institute of Agrifood Research and Technology) facilities in La Ràpita. Prior to the experiments, they were acclimated in 2000 L experimental tanks, with two individuals per tank, each kept in separate compartments divided by mesh partitions. During an acclimation period of one week, octopuses were fed dead blue crabs. A total of 15 common octopuses (males and females ~50% each) were included in the experiments. Trials with subadult octopuses (*n* = 9) were conducted between 29 October and 10 November 2021, under an average water temperature of approximately 18.0 °C and a salinity of 36.5. Trials with adult octopuses (*n* = 6) were carried out between 19 April and 20 May 2022, under similar environmental conditions. In all experiments, environmental parameters were carefully controlled: dissolved oxygen saturation remained above 95%, pH was stable, and a natural photoperiod regime was maintained.

A total of 19 loggerhead sea turtles were included in the study. All individuals were rescued by the Oceanogràfic Foundation (València, Spain), as part of the Valencian Community Stranding Network during 2022 and 2023, mostly after incidental capture by trawlers or gillnets. All tests were conducted once the individuals were discharged by the veterinary service, prior to be released back to the sea. During their rehabilitation, turtles were regularly fed with natural prey (crabs, mollusks, and fish), minimizing potential alterations in foraging behavior. The experimental group included approximately 60% females and 40% males, a proportion that remained consistent across both size preference and biomass consumption trials. Sea turtles were maintained in individual tanks (3000 to 30,000 L) at 23 °C and salinity of 37.5, and were acclimated with dead blue crab for one week before the trials. Dissolved oxygen saturation was maintained above 95%, pH remained stable, and tanks followed a natural photoperiod regime. Individuals were categorized into three size classes based on curved carapace length (CCL): juvenile (T_J, CCL up to 59.9 cm), subadult (T_SA, CCL range 60–69.9 cm), and adult (T_A, CCL > 70 cm) [14,34,35,36].

A limited number of individuals were reused in multiple trials due to animal welfare restrictions and availability constraints. Although resting and fasting periods were applied between trials, we acknowledge that observations from the same predator are not fully independent, representing a risk of pseudo replication that is accounted for in the interpretation of results.

Blue crabs used in the experiments were obtained from commercial fisheries in Albufera of Valencia and the Ebro Delta. Crabs were kept alive and transferred to either Oceanogràfic (for sea turtles’ trials) or IRTA La Ràpita (for octopuses’ trials) facilities. They were classified into four size classes: small (S, ca. 140 g), medium (M, 220 g), large (L, 330 g), and extra-large (XL, 450 g).

### 2.2. Size Preference Experiments

For each trial, a single predator was offered a simultaneous combination of three crab sizes depending on seasonal availability. Subadult octopuses (O_SA) were presented with S, L, and XL crabs (*n* = 12), while adult octopuses (O_A) were offered S, M, and L crabs (*n* = 6). Loggerhead sea turtles of juvenile (T_J, *n* = 6), subadult (T_SA, *n* = 6), and adult (T_A, *n* = 7) sizes were also presented with a combination of S, M, and L crabs (see Table 1 for details). All trials were conducted following a 24 h fasting period.

A few individuals were reused within the same type of experiment—specifically, three subadult octopuses and one juvenile, two subadult, and two adult sea turtles. In these cases, a minimum rest period of 24 h for octopuses and 48 h for sea turtles was applied, followed by an additional 24-h fasting period prior to the next trial.

The order in which each predator captured and consumed crabs of different sizes was continuously monitored until all prey were consumed or 24 h had elapsed. To facilitate this process, trap cameras were installed to record the entire sequence of prey selection and capture. For octopuses, time to capture (in minutes) was also recorded.

### 2.3. Maximum Biomass Consumption Experiments

Each predator was provided with a weighed, ad libitum supply of crabs for 24 h. The same individuals used in the size preference experiments were subsequently used in the biomass consumption trials.

For octopuses, biomass consumption was assessed for each combination of predator and crab size (*n* = 6 per combination) (Table 1). In the case of the subadult specimens, 6 individuals were randomly selected from the 9 available for each size combination, while for adult specimens, the same 6 individuals were used for all three biomass trials with different crab sizes. Individuals were reused following a 24-h rest period and a subsequent 24-h fasting period before the next trial.

For each loggerhead sea turtle size class, between 9 and 14 replicate trials were conducted (T_J, *n* = 12; T_SA, *n* = 9; and T_A, *n* = 14), in which a mixture of crabs of different sizes was offered (Table 1). In this case, both new individuals and those previously used in the size preference experiments were included in the biomass consumption trials. For the reused individuals, a 48-h rest period followed by a 24-h fasting period was applied.

In the case of the experiments with sea turtles, the consumed biomass of blue crab was expressed as total biomass (TB in gWW·d^−1^) as the entire crab was consumed. In contrast, for octopuses, blue crab biomass data include only the soft tissue portion (STB in gWW·d^−1^), as these predators do not consume the exoskeleton. The proportion of soft tissues relative to total crab biomass was also estimated (*n* = 5).

### 2.4. Data Analysis

All data are presented as the mean ± standard deviation (SD). Morphometric (wet weight (WW) for all organism types, as well as curved carapace length (CCL) for loggerhead sea turtles and carapace width (CW) for blue crabs) and biomass intake data were tested for normality and homogeneity of variances (Levene’s test), and log10(x) transformed if necessary. One-way ANOVAs and Tukey post hoc tests were used for normally distributed data. For non-normal data, Kruskal-Wallis and Bonferroni post hoc tests were applied (Statgraphics Centurion XVII). The level of statistical significance was set at *p* < 0.05.

For each of the food size preference experiments (Octopus: rank order of capture and consumption time for 2 predator sizes; sea turtles: rank order of capture for 3 predator sizes) Friedman ANOVAs by ranks [37] and Kendall’s concordance coefficient [38] were used to assess differences in preference between crab sizes (S, M and L with the exception of S, L and XL for O_SA) and the degree of agreement among the rankings (see Prado & Heck [39] and Camps-Castellà et al. [40] for a similar approach). In addition, non-parametric post hoc comparisons (Wilcoxon Matched Pairs test) were conducted when significant differences were observed. For each size preference experiment, crabs (S/M/L or S/L/XL) were assigned a 1 to 4 score based on the order of preference in which each crab was captured and consumed (totally or partially): (1) first choice, (2) second choice, (3) third choice, (4) not consumed. With the octopuses, the time to capture (min) of each crab size was also assessed, but an arbitrary value of 1500 min ≡ 25 h (i.e., greater than the experimental time of 24 h) was assigned when the crab was not consumed. STATISTICA v.12 software was used for all these rank order and time analyses.

## 3. Results

### 3.1. Predator and Prey Characteristics, and Feeding Strategies

The weight of the subadult (O_SA) and adult (O_A) octopuses used in the experiments was significantly different, with mean values of 1183 ± 169 g and 2592 ± 337 g, respectively (ANOVA, *p* < 0.001). Loggerhead sea turtles also showed significant differences among the three size classes (juveniles, subadults, adults) in both body weight and curved carapace length (Kruskal-Wallis, *p* < 0.001). Juveniles (*n* = 8) had a mean weight of 11,848 ± 3884 g and a CCL of 45.3 ± 5.7 cm, subadults (*n* = 5) 28,660 ± 3715 g and 61.7 ± 1.9 cm, and adults (*n* = 6) 71,783 ± 9340 g and 80.0 ± 4.1 cm.

Blue crabs used as prey were classified into four size classes based on wet weight (WW) and carapace width (CW): small (S, 142 ± 29 gWW; 136 ± 11 mm CW), medium (M, 215 ± 38 gWW; 161 ± 11 mm CW), large (L, 327 ± 62 gWW; 190 ± 14 mm CW), and extra-large (XL, 448 ± 31 gWW; 216 ± 6 mm CW) (Kruskal-Wallis test applied to WW and CW, *p* < 0.001). The proportion of soft tissues relative to total crab biomass was 53.9 ± 9.6%.

**Figure 1:**
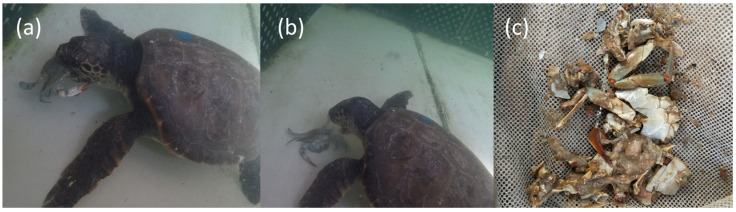
Predation and digestion of *C. sapidus* by *C. caretta*. (**a**) Capture by biting. (**b**) The sea turtle releases the blue crab, revealing a visible bite mark on the carapace. (**c**) Sea turtle fecal remains showing undigested chitin fragments and abundant mucus.

**Figure 2:**
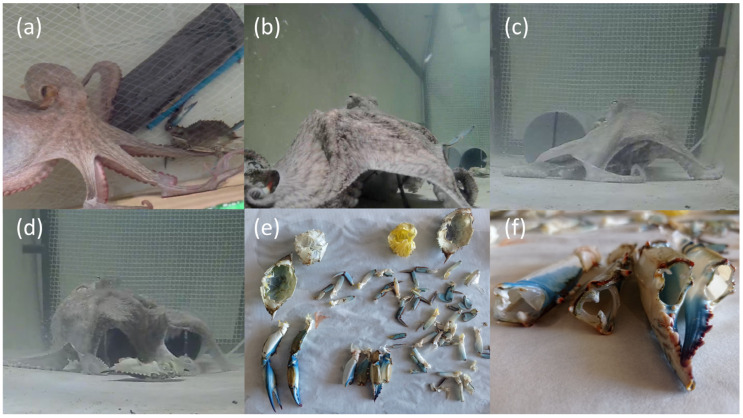
Predation sequence of *C. sapidus* by *O. vulgaris*. (**a**) Blue crab capture with extended arms. (**b**,**c**) Immobilization and ventral opening of the cephalothorax. (**d**) Hard exoskeletal parts, cleaned of soft tissues, are expelled outside the interbrachial membrane. (**e**) Remains of blue crab exoskeletons after feeding. (**f**) Chelipeds completely emptied of muscle tissue.

Loggerhead sea turtles ingested the crabs whole, including the exoskeleton, which they were able to crush using their strong keratinized beak (Figure 1). In contrast, octopuses immobilized the crabs with their arms, enveloped them with the interbrachial membrane, then lifted the carapace, and dismembered to consume the soft internal tissues, leaving the exoskeleton behind (Figure 2). This difference in feeding strategy is reflected in the biomass metrics used: total biomass (TB) for loggerhead sea turtles and soft tissue biomass (STB) for octopuses.

### 3.2. Size Preference Experiments

In octopuses, no significant differences were observed in the order of crab size selection during the preference trials (Table 2). For subadult individuals (O_SA), the scores for size order were 1.8 ± 1.0 (S), 2.2 ± 1.2 (L), and 2.9 ± 1.3 (XL), showing no statistically significant preference (χ^2^ = 3.17, *p* = 0.205), although a tendency toward lower selection of XL crabs was observed. Capture time was significantly shorter for small crabs (225 ± 449 min) compared to large (479 ± 635 min) and extra-large (926 ± 630 min) crabs (χ^2^ = 9.43, *p* = 0.009). In adult octopuses (O_A), neither capture order nor time differed significantly among crab sizes.

Among loggerhead sea turtles, no statistically significant size preferences were detected within each size class. However, when data were pooled across all turtles (T_All), a near-significant trend emerged: small crabs were chosen first (1.8 ± 1.2), followed by medium (2.1 ± 1.1) and large (2.6 ± 1.1) crabs (χ^2^ = 5.15, *p* = 0.076; Table 3).

### 3.3. Biomass Consumption

**Figure 3:**
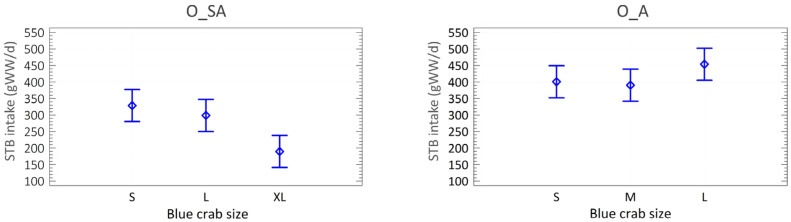
Soft Tissue Biomass (STB) intake (gWW·d^−1^) of the 3 crab sizes consumed by subadult (O_SA) and adult (O_A) octopuses. Mean and LSD (Fisher’s least significant difference) intervals.

Adult octopuses consumed an average of 415 gWW·d^−1^ of soft tissue biomass (STB), with similar consumption across small (401 ± 31), medium (390 ± 113), and large crabs (454 ± 71; ANOVA, *p* = 0.3547). In subadults, daily consumption averaged 272 STB gWW·d^−1^, with significant differences among crab sizes: consumption was higher for small (329 ± 88) and large (299 ± 90) crabs than for extra-large ones (190 ± 51; ANOVA, *p* = 0.0193; Figure 3).

Loggerhead sea turtles consumed the entire crab, and their total biomass intake (TB) varied significantly among size classes (Table 4). Juveniles consumed 197 ± 114 TB gWW·d^−1^, while subadults and adults consumed significantly higher amounts: 627 ± 554 and 815 ± 592 TB gWW·d^−1^, respectively (ANOVA F = 8.67, *p* = 0.0010).

## 4. Discussion

### 4.1. Feeding Behavior and Consumption Rates

Our experimental results demonstrate that both the common octopus and the loggerhead sea turtle are capable of consuming substantial amounts of the invasive blue crab under controlled conditions. However, these two native Mediterranean predators differ markedly in their feeding strategies, prey handling techniques, and overall efficiency.

Octopuses exhibited specialized feeding behavior, targeting only the soft tissues of the crabs and discarding the exoskeleton. These observations are consistent with the feeding pattern described by Guerra [18]. *Octopus vulgaris* is an opportunistic predator and scavenger with a broad diet including crustaceans, mollusks, and fish [19,20]. This behavior has also been observed with *Callinectes sapidus*, both in controlled experiments [30] and in the wild along Mediterranean coasts, where the remains of blue crabs have been documented near octopus’ dens [12] and photographic and video evidence of this predator-prey interaction have been captured [13].

In our trials, adult octopuses (average body weight: 2592 g) consumed a mean of 415 g of crab soft tissue biomass (STB) per day, equivalent to approximately 16% of their own body weight, whereas subadult individuals (average 1183 g) consumed 272 g per day, corresponding to 23% of body weight. These values are notably high, exceeding the range of 10–20% of body weight per day typically reported for young octopuses, and well above the 2–5% observed in later life stages [41]. They also surpass the absolute feeding rates of 100 g per day estimated under aquaculture conditions with crabs [42]. Food intake in octopuses is known to depend on several factors including temperature, predator size, prey availability, and food quality [41,42]. Intake is particularly high when crabs are offered alive [18], which triggers the predatory instinct and reinforces the high feeding capacity of this species. It should be noted, however, that the exceptionally high percentages observed in this study may have been partly influenced by the experimental design, which involved a fasting period prior to trials; under non-fasting conditions, food intake would likely be somewhat lower.

No significant differences in consumption were found across small, medium, and large crab sizes in adult octopuses. However, subadult octopuses showed significantly lower intake when presented with extra-large crabs (~450 g), likely due to the increased energetic cost and difficulty of handling prey that approaches half of their own body weight (~1 kg). It is also worth noting that these values refer only to soft tissue biomass, which represented approximately 54% of total crab biomass in our study. Thus, in terms of potential biomass removal from the ecosystem, octopus’ consumption would nearly double the measured STB.

In contrast, loggerhead sea turtles consumed the entire crab, including the chitinous exoskeleton. This ability to crush and ingest hard-bodied prey is well documented in both Mediterranean [14,25,43] and in Atlantic populations, where *C. sapidus* is native and commonly found in loggerhead diets [27,44,45]. In our study, total biomass (TB) ingestion rates reached 197 gWW·d^−1^ in juveniles and exceeded 600–800 gWW·d^−1^ in subadults and adults. Notably, juvenile turtles (~11.8 kg) slightly exceeded the maximum daily intake of 149–172 g/day reported by Swingle et al. [46] for individuals under 20 kg. These results underscore the capacity of *Caretta caretta* to achieve high feeding rates when blue crabs are abundant.

Furthermore, our findings are in agreement with energy requirement models. Njoman et al. [47] estimated that, at ~20.5 °C, juvenile loggerheads reach basal metabolism at a daily intake of 1.01% of body weight. In our study, food consumption reached 1.67% for juveniles, 2.19% for subadults, and 1.14% for adults—values that suggest not only maintenance but also potential for growth. Similarly, Valente et al. [48] reported experimental feeding rations equivalent to 1.5–2.5% of body mass in juvenile and subadult turtles, which fall within the range observed in our trials.

Nonetheless, despite the ability of sea turtles to ingest high volumes of prey, fecal analyses revealed abundant undigested chitin fragments and mucus in turtle excreta, which may suggest irritation or inflammation of the digestive mucosa. Similar pathologies have been described by Inurria et al. [49], who reported gastrointestinal lesions and mortality in *C. caretta* associated with the ingestion of large amount of sea urchins—another prey type with hard, indigestible parts. This raises concerns about the cost-benefit balance of repeatedly consuming hard-shelled prey such as *C. sapidus*, particularly under conditions of low dietary diversity.

Together, these findings confirm the strong predatory potential of both species, with octopuses showing high consumption rates of digestible tissues and sea turtles demonstrating their ability to ingest whole crabs in significant quantities—albeit frequent ingestion of hard prey may impose some digestive disruptions.

Neither predator exhibited a statistically significant preference in the order of crab size selection during the trials. However, subadult octopuses required significantly less time to capture small crabs than larger ones, possibly reflecting reduced handling time and effort. While the order of capture did not differ significantly among sizes, a slight tendency to avoid larger crabs was observed, particularly in subadult octopuses (Table 2). This pattern, resulting from a functional preference shaped by both predator traits (e.g., inclination to target smaller prey) and prey properties (e.g., defensive behavior), should be interpreted with caution given the limited number of predators available in the trials. Similar findings on size-related handling strategies were reported by Grisley et al. [50], who found that octopus species modified their attack strategies depending on crab size, with larger prey requiring more complex manipulation. Loggerhead sea turtles also showed a near-significant trend toward selecting smaller crabs when data were pooled, likely due to the lower difficulty in attacking and subduing less defensive individuals. Nonetheless, both predators successfully captured and consumed even the largest crabs offered, highlighting their capacity to handle a wide size range of this invasive prey. Possible sex-related effects could not be assessed in this study, but the balanced proportions of males and females reduced the likelihood of bias. These patterns are best interpreted as functional preferences, since the observed trends likely reflect the combined influence of predator behavior and prey acceptability under our choice-only experimental design.

### 4.2. Ecological Implications and Management Perspectives

The outcomes of our laboratory trials align with recent field observations. *O. vulgaris* has been repeatedly observed preying on *C. sapidus* in the wild along the Mediterranean coast [12,13], suggesting an adaptive response to the increasing availability of this invasive prey. Consistent with this, *C. sapidus* has been detected in the gastrointestinal contents of loggerhead sea turtles from the Adriatic Sea [14], confirming that both predators interact with this species in natural Mediterranean habitats and could play a role in its control.

Although both predators interact with *C. sapidus* in Mediterranean habitats, their potential impact on invasive populations is unlikely to be equivalent. The common octopus is considerably more abundant in shallow coastal ecosystems than loggerhead sea turtles [51,52,53], where it represents one of the most important benthic predators [22,54]. This greater numerical presence, combined with its generalist diet and high feeding rates, suggests that *O. vulgaris* may exert a stronger predation pressure on blue crab populations than *C. caretta*, whose contribution is more episodic and dependent on spatial overlap with crabs.

The seasonal life cycle of *C. sapidus* may further enhance its susceptibility to being captured by native predators. In systems such as L’Albufera de Valencia, reproductive migrations of ovigerous females from estuarine to coastal marine waters peak in July to support larval development in high-salinity environments [55]. This movement into shallow coastal zones—where *O. vulgaris* is commonly found [16,22] —may increase predator-prey encounters during a vulnerable stage of the crab’s life cycle, for adult females. Such phenological overlap could amplify the biocontrol potential of native octopus’ populations.

The potential role of *O. vulgaris* in controlling other invasive species, such as the lionfish (*Pterois miles*) [56] has already been recognized in the Mediterranean Sea, reinforcing the broader concept of integrating native predators into invasive species management [57]. *C. caretta* is also capable of consuming substantial quantities of *C. sapidus*; however, its contribution to population control may be more localized, depending on spatial overlap and individual foraging behavior. Protection and recovery of loggerhead turtle populations may therefore contribute to reinforcing natural top-down control mechanisms, especially in high-density invasion zones.

Our results provide mechanistic insights into predator-prey interactions that may contribute to natural biocontrol of *C. sapidus* in the Mediterranean. However, they should be interpreted with caution, as feeding motivation, predator behavior, and prey encounter rates in captivity may differ from natural conditions—particularly in the case of rehabilitated sea turtles, whose diet and foraging responses may be temporarily altered during recovery. In addition, the exclusive use of blue crabs as available prey may have enhanced predatory responses compared to those expected in a more diverse natural prey field.

These findings highlight the importance of preserving native predator populations and incorporating trophic interactions into integrated management frameworks. Supporting robust populations of *O. vulgaris* and *C. caretta* through conservation and fishery regulations may offer a cost-effective and ecologically sound approach to mitigating the impact of blue crab invasions in Mediterranean ecosystems.

## 5. Conclusions

This study demonstrates that both the common octopus and the loggerhead sea turtle can act as effective predators of the invasive Atlantic blue crab in the Mediterranean. Octopuses exhibited high consumption rates by selectively feeding on soft tissues, whereas turtles ingested whole crabs across size classes. The common octopus is the predator most likely to exert stronger control over blue crab populations, not only because of its higher effective consumption rates but also due to its widespread abundance along Mediterranean coasts. However, the extent to which these experimental results translate into effective biocontrol under natural conditions remains uncertain and should be further evaluated through field studies. Our findings indicate that neither adult crab size class fully prevents predation by *O. vulgaris* or *C. caretta* under experimental conditions, although smaller crabs tended to be more vulnerable. These findings provide valuable insights into the potential role of native predators in helping regulate invasive blue crab populations and supports their consideration within future integrated management strategies.

## Figures and Tables

**Table 1:** Experimental trials with octopuses (O) and loggerhead sea turtles (T). Predator size classes: juveniles (J), subadults (SA), and adults (A). Blue crab size categories: S (small), M (medium), L (large), and XL (extra-large). The table summarizes the number of predators of each size class used, the trials performed by predator size class, and the crab size combinations used in each type of experiment (size preference and maximum daily biomass consumption). For sea turtles, the number of trials per crab size combination in the maximum biomass consumption experiments is also shown.

Predator Species	Experiment Type	Predator Size	No. of Predators	No. of Trial (*n*)	No. of Trials/Crab Size Combination (*n*)	Blue Crab Sizes			
*Octopus vulgaris*	Size preference	O_SA	9	12		S		L	XL
		O_A	6	6		S	M	L	
	Maximum daily biomass	O_SA	6	6		S			
			6	6				L	
			6	6					XL
		O_A	6	6		S			
			6	6			M		
			6	6				L	
*Caretta caretta*	Size preference	T_J	5	6		S	M	L	
		T_SA	4	6		S	M	L	
		T_A	5	7		S	M	L	
	Maximum daily biomass	T_J	8	12	4	S	M	L	
					2	S	M		
					1		M	L	
					3		M		
					1			L	
					1	S			
		T_SA	5		5	S	M	L	
				9	3		M	L	
					1		M		
		T_A	6	14	4	S	M	L	
					1		M	L	
					3		M		
					6			L	

**Table 2:** Size preference experiments of crabs with subadult (O_SA) and adult octopuses (O_A). Blue crab sizes: S (small), M (medium), L (large) and XL (extra-large). Order (score according to the order of preference in which it was captured: 1_first choice, 2_second choice, 3_third choice, 4_not consumed), Time (time to capture in minutes). Mean ± SD, Friedman’s ANOVA (χ^2^) and Kendall’s coefficient of concordance (*W*). In the same row, different superscript letters indicate significant differences in Wilcoxon matched pairs post hoc comparisons.

		S	M	L	XL	Friedman’s ANOVA χ^2^	Kendall’s *W*	*p*-Value
**O_SA** (*n* = 12)	Order	1.8 ± 1.0		2.2 ± 1.2	2.9 ± 1.3	3.17	0.132	0.205
	Time	225 ^a^ ± 449		479 ^b^ ± 635	926 ^b^ ± 630	9.43	0.493	**0.009**
**O_A** (*n* = 6)	Order	2.0 ± 1.1	2.2 ± 1.5	2.3 ± 1.5		0.11	0.009	0.949
	Time	392 ± 581	560 ± 741	647 ± 688		0.95	0.079	0.623

**Table 3:** Size preference experiments of crabs with juvenile (T_J), subadult (T_SA), adult (T_A) and the set of all sizes (T_All) loggerhead sea turtles. Blue crab sizes: S (small), M (medium) and L (large). Score according to the order of preference in which the crab was captured (1_first choice, 2_second choice, 3_third choice, 4_not consumed). Mean ± SD, Friedman’s ANOVA (χ^2^) and Kendall’s coefficient of concordance (*W*).

	S	M	L	Friedman’s ANOVA χ^2^	Kendall’s *W*	*p*-Value
**T_J** (*n* = 6)	2.2 ± 1.6	2.0 ± 1.2	3.2 ± 1.1	2.00	0.200	0.368
**T_SA** (*n* = 6)	1.4 ± 0.6	2.2 ± 1.1	2.6 ± 1.3	1.63	0.163	0.444
**T_A** (*n* = 7)	1.9 ± 1.2	2.1 ± 1.1	2.3 ± 0.8	1.92	0.137	0.382
**T_All** (*n* = 19)	1.8 ± 1.2	2.1 ± 1.1	2.6 ± 1.1	5.15	0.151	0.076

**Table 4:** Total biomass intake (TB gWW·d^−1^) by different loggerhead sea turtle classes (juvenile (T_J), subadults (T_SA) and adults (T_A)). Mean ± SD. One-way ANOVAs, Tukey post hoc comparison. In the same row, different superscript letters indicate significant differences.

	T_J	T_SA	T_A	F-Ratio	*p*-Value
***n***	12	9	14		
**TB intake (gWW**·**d^−1^)**	197 ^a^ ± 114	627 ^b^ ± 554	815 ^b^ ± 592	8.67	**0.0010**

## Data Availability

Data available in a publicly accessible repository. The data presented in this study are openly available at doi:10.4995/Dataset/10251/226705.
